# Letter and Receipt from Mrs. Kilpatrick

**Published:** 1888-01

**Authors:** 


### PHYSICIANS' MUTUAL BENEFIT ASSOCIATION OF TEXAS.

The Secretary of this Association, Dr. Daniel, mailed the following communication to Mrs. A. R. Kilpatrick on the 1st of January, inst.:

OFFICE PHYSICIANS' MUTUAL BENEFIT ASSOCIATION OF TEXAS.

AUSTIN, TEXAS, January 1, 1888.

MRS. A. R. KILPATRICK,

Navasota, Texas.

DEAR MADAM:—As Secretary and Treasurer of the Physicians' Mutual Benefit Association of Texas, I hand you herewith sight draft on Ball, Hutchins & Co., Galveston, for $48.70, being the balance due you on assessment. This sum, with the $95.00 sent you previously, makes $143. 70, the total amount collected from 148 members, less $4.30 paid for postal cards, printing same, and stamps on receipts, of which find memorandum inclosed.

I am mortified to say that nine members of the one hundred and fifty-seven who were in good standing at the date of Dr. Kilpatrick's death, and on whom assessment was made, failed, for some reason, to respond; and hence forfeited their membership, and are dropped. In the majority of cases I must believe it was unintentional, and due to absence or removal, or failure to receive notice; for some who have, at this late date, just sent in their contributions state that they did not receive the first notice, and it is probable that others still failed to receive my second notice.

In the January number of the Journal the names of all the members who responded to the assessment , will be published in full.

As is my duty, in the name and on behalf of the Physicians' Mutual Benefit Association of Texas, I respectfully ask you to accept this small sum of money—a free will offering of a devoted little band of physicians who acknowledged and held in high appreciation your lamented husband's many excellent qualities of head and heart, as a feeble expression of their sympathy with you in his loss from the ranks of medicine, as well as from the domestic hearth. With kinds regards, believe me Sincerely yours,

F. E. Daniel,
Secretary.

Below will be found the list of members who paid their *pro rata* of the above sum; together with Mrs. Kilpatrick's receipt for $143,-70, inclosed in the following note:

Navasota, Texas, January 7, 1888.

*Dr. F. E. Daniel.*

Dear Sir:—Your's received a few days since. Please excuse delay. I thank you and the members of the Association who have so kindly assisted me in my time of need.

It shall ever be a pleasant duty with me to say what I can to add to your numbers.

I inclose the receipt, with great appreciation of your kindness.

Respectfully,
Mrs. A. R. Kilpatrick.

$143.70

Received of F. E. Daniel, Secretary and Treasurer of the Physicians' Mutual Benefit Association of Texas, ninety-five dollars (receipted for previously) and January 3, 1888, forty-eight dollars and seventy cents, in full one hundred and forty-three dollars and seventy cents. Mrs. F. L. Kilpatrick.

Navasota, Texas, January 5, 1888.

